# Micro- to macroscale magnetic resonance imaging of glioma

**DOI:** 10.1007/s10334-021-00999-w

**Published:** 2022-02-02

**Authors:** Patricia Clement, Marion Smits, Matthias J. P. van Osch, Bruno M. Costa, Esther A. H. Warnert

**Affiliations:** 1 Ghent Institute for Functional and Metabolic Imaging (GIfMI), Ghent University, Ghent, Belgium; 2 Department of Radiology & Nuclear Medicine, Erasmus MC, Rotterdam, The Netherlands; 3 Erasmus MC Cancer Institute, Erasmus MC, Rotterdam, The Netherlands; 4 C.J. Gorter Center for High Field MRI, Department of Radiology, Leiden University Medical Center, Leiden, The Netherlands; 5 Leiden Institute for Brain and Cognition (LIBC), Leiden, The Netherlands; 6 Department of Radiology, Leiden University Medical Center, Leiden, The Netherlands; 7 School of Medicine, Life and Health Sciences Research Institute (ICVS), University of Minho, Braga, Portugal; 8 ICVS/3Bs-PT Government Associate Laboratory, Braga, Guimaraes, Portugal

In the European Union, approximately 44,000 patients are diagnosed with glioma each year [1]. Adult-type glioma is classified according to the World Health Organisation (WHO) based on a combination of histopathological and molecular features, including co-deletion of 1p/19q chromosome arms and isocitrate dehydrogenase mutational (IDH) status [2]. With no existing cure and overall median survival ranging from > 10 years for IDH mutated, 1p/19q co-deleted lower grade glioma to only 15 months for the most aggressive form of glioma (IDH wild-type, glioblastoma), tremendous research efforts are being made to increase understanding and depiction of glioma pathology and improve treatment strategies with associated monitoring of treatment outcome. As a widely available and non-invasive medical imaging modality for investigating the structure and (patho)physiology of the brain, MRI should play a key role in these research efforts.

During the last decades, the scale of MRI in research and clinical application has simultaneously become smaller and larger. Constant advances in MR hardware, acquisition, reconstruction, and image analysis have enabled microscale measurements, including quantitative imaging of molecular and cellular processes and assessing genetic information with radiomics. Simultaneously, the increased number of potential contrasts to acquire, together with accelerated data acquisition schemes, have led to a norm of increasingly multiparametric MRI data acquisition in research and clinical settings. Moreover, the urge to harmonize data acquisition and the rise of machine and deep learning have led to the current drive for international, multicenter and multidisciplinary collaboration and data integration to generate big and truly meaningful imaging datasets.

The European COST Action Glioma MR Imaging 2.0 (GliMR^1^) is an international network founded in September 2019 and aims to unite experts in multiple disciplines, including MRI physicists and engineers, molecular biologists, computer scientists, neuroradiologists, and treating physicians, to promote the micro- to macroscale potential of MRI to advance imaging diagnostics of glioma. This ranges from in vivo imaging of glioma pathophysiology at the molecular level to the development of deep learning algorithms allowing for multi-site, multi-vendor MR image analyses to aid non-invasive glioma diagnosis, treatment planning, and follow-up based on big, heterogeneous datasets. This special issue of Magnetic Resonance Materials in Physics, Biology and Medicine aims to provide a cross section of the ongoing research on advanced MRI acquisition and analysis techniques that aim to further glioma imaging diagnostics from improved understanding of the molecular pathophysiology, via increased accuracy of diagnosis, to reaching optimized, patient-centered treatment planning and follow-up.

In this special issue, novel imaging approaches can be found that target differences in molecular physiology between tumor and healthy tissue. Ebrahimpour et al. illustrate the ability to use quantitative imaging to visualize increased iron uptake, mediated by 5-aminolevulinic acid, in vitro and in a rat model of glioblastoma. Assessing microvascular structure and function, beyond the traditionally used dynamic susceptibility contrast (DSC) or dynamic contrast enhanced (DCE) imaging, is another area of active research to improve tumor localization and delineation. Paschoal et al. and Stumpo et al. present novel approaches exploiting the IVIM (intravoxel incoherent motion) contrast and cerebrovascular reactivity measurements to investigate microvascular structure and function of glioma.

Moreover, when novel biomarkers for identification of glioma have been identified, a next step is to properly explore those biomarkers toward clinical use. Juskanic et al., Warnert et al., Wu et al., and Lindig et al. show in this special issue that novel molecular imaging-based biomarkers can be used to explicitly visualize non-enhancing glioma, differentiate IDH wild-type from IDH mutant tumors, and to measure glucose metabolism within glioma. In particular the latter is an emerging technique, of which the current state of the art is neatly reviewed by Golay et al. in this special issue. All of these works illustrate the potential of using advanced imaging techniques in the clinical, diagnostic work-up for patients diagnosed with glioma.

An important aspect for clinical adoption of novel imaging techniques to be used for diagnosis and, in particular, disease monitoring, is illustrating the reproducibility of this method. In this special issue, Kleppestø et al. investigate the use of population-based arterial input functions to improve the inter-operator repeatability for DCE MRI in high grade gliomas. The increasing call for gadolinium-free imaging has given rise to the use of arterial spin labeling (ASL) to assess glioma perfusion, rather than the traditional DSC/DCE MRI. Alsaedi et al. herein illustrate that glioma perfusion can be assessed with ASL in a repeatable manner.

The rise of advanced MRI to assess tumor physiology and better visualize and characterize microscopic invasion is paving the way for the use of biomarkers arising from these techniques to improve upon radiotherapy treatment planning of gliomas. An overview of such advanced biomarkers in the current literature is given by Tang et al.

In recent decades, progression-free survival in patients diagnosed with glioma has increased because of increased understanding of tumors and improved treatment strategies. As a result, long-term effects of treatment on normal-appearing brain tissue are a growing problem and research is ongoing to use quantitative MRI techniques to assess damage to the (assumed) healthy brain areas. Within this special issue, this is specifically addressed for gray matter atrophy by Gommlich et al., perfusion and diffusion MRI by Ertekin et al., while a systematic review of the latest literature encompassing a range of quantitative MRI techniques for this purpose is given by Petr et al.

GliMR continues to build upon the work and collaborations presented within this special issue to further improve glioma diagnosis, and treatment planning and follow-up by facilitating multicenter and international collaborations across Europe. For more information on how to join our network and activities visit www.glimr.eu.

## Footnotes

1 GliMR is funded by the European Cooperation in Science and Technology, www.cost.eu.
